# Total and Free Sugar Levels and Main Types of Sugars Used in 18,784 Local and Imported Pre-Packaged Foods and Beverages Sold in Hong Kong

**DOI:** 10.3390/nu13103404

**Published:** 2021-09-27

**Authors:** Lok Yin Chan, Daisy H. Coyle, Jason H. Y. Wu, Jimmy Chun Yu Louie

**Affiliations:** 1 Faculty of Science, School of Biological Sciences, The University of Hong Kong, Pokfulam, Hong Kong 999077, China; chanlogin@gmail.com; 2 Food Policy Division, The George Institute for Global Health, Camperdown, NSW 2050, Australia; dcoyle@georgeinstitute.org.au (D.H.C.); jwu1@georgeinstitute.org.au (J.H.Y.W.)

**Keywords:** sugars, free sugars, pre-packaged foods, Hong Kong, method, added sugars

## Abstract

There is limited information regarding the free sugar content of pre-packaged foods in Hong Kong. This study aims to assess the free sugar content and identify the most frequently used free sugar ingredients (FSI) in pre-packaged foods in Hong Kong. Data from 18,784 products from the 2019 FoodSwitch Hong Kong database were used in this analysis. Ingredient lists were screened to identify FSI. Total sugar content was derived from nutrition labels on packaging. Free sugar content was estimated based on adaptation of a previously established systematic methodology. Descriptive statistics of the total sugar and free sugar content, as well as the mean ± SD contribution of free sugar to total sugar of the audited products were calculated, stratified by food groups. Almost two-thirds (64.5%) of the pre-packaged foods contained at least one FSI. ‘Sugar (sucrose)’ was the most popular FSI that was found in more than half (54.7%) of the products. ‘Fruit and vegetable juices’ (median 10.0; IQR 8.3–11.5 g/100 mL) were found to have a higher median free sugar content than ‘Soft drinks’ (8.0; 6.0–10.6 g/100 mL). Mean ± SD contribution of free sugar to the total sugar content was 65.8 ± 43.4%, with 8 out of 14 food groups having >70% total sugar as free sugar. To conclude, free sugar, especially sucrose, was extensively used in a wide variety of pre-packaged products sold in Hong Kong. Further studies are needed to assess the population intake of free sugar in Hong Kong to inform public health policy on free sugar reduction.

## 1. Introduction

Free sugars are often called ‘empty calories’ as they provide energy with little or no nutritional value [1]. Excessive intake of free sugars is associated with common health problems including obesity [2], cardiovascular diseases [3,4], diabetes [5], and dental caries [6]. As a result, the World Health Organization (WHO) has strongly recommended a maximum intake of free sugars to less than 10% of total energy intake; with a conditional recommendation of below 5% for additional health benefits [7]. The stricter cut-off of 5% was also recommended for the child population by other public health groups such as the European Society for Paediatric Gastroenterology, Hepatology and Nutrition (ESPGHAN) [8] and the UK Scientific Advisory Committee on Nutrition (SACN) [9].

In Hong Kong, half of those aged 15–84 years are either obese or overweight (50.1%) [10]. Furthermore, more than half of all 5-year-olds have experienced tooth decay [11], and around 10% of the whole population suffers from type 2 diabetes mellitus, the tenth most common cause of death in Hong Kong [12]. Although there are no official statistics regarding the sugar intake of the Hong Kong population, based on usual dietary habits and the high availability and accessibility of sugary products in the food supply, the sugar consumption of Hong Kong citizens is believed to exceed the Chinese Dietary Reference Intake for added sugar of <10% total energy intake [13] by a considerable margin. In response to this and the WHO sugar guidelines, the Hong Kong government has introduced several interventions to promote reduction in the population’s sugar intake, such as social media campaigns, health promotion events, as well as a voluntary front-of-package labelling scheme [14,15,16]. Under the voluntary front-of-package labelling scheme, eligible products can display a front-of-package logo to highlight their low (<5 g per 100 g or 100 mL) or no (<0.5 g per 100 g or 100 mL) sugar content. Nonetheless, the uptake of this scheme is low due to its voluntary nature [17].

For pre-packaged products, consumers can only rely on the nutrition information panel on the package to evaluate the nutrition composition of a product. Unlike in the United States, which now requires that all pre-packaged products display the added sugar content on the nutrition label [18], in Hong Kong, only the total sugar content, which includes both naturally occurring (intrinsic) sugars and free sugars, has to be declared on the nutrition labels [19]. This means that consumers can only determine whether a product contains free sugar by identifying free sugar ingredients (FSI) from the ingredient list. However, given that many different types of FSI are found in the food supply [20,21], this may prove challenging for some consumers. 

Numerous types of FSI are used by manufacturers including sucrose, high-fructose corn syrup (HFCS), honey, and glucose [21,22]. Each sugar is metabolized differently after consumption and therefore may lead to different health effects [23]. For instance, dietary fructose and HFCS seem to have a closer association with obesity and metabolic syndrome when compared with glucose [24,25]. Thus, the main types of free sugars used in the pre-packaged food supply could impact public health, although this has not yet been extensively investigated.

Currently, there is limited data on the free sugar content of the pre-packaged food supply in Hong Kong, as this information is not displayed on packaging, and there are no analytical methods to distinguish between free sugars and naturally occurring sugars, which are chemically identical [26,27]. The aims of this study are therefore to assess the free sugar content of pre-packaged foods and beverages in Hong Kong by modifying a step-by-step decision algorithm [22,28] to compare free sugar levels across major and minor food categories, and to identify the major types of free sugar present in pre-packaged food supplies.

## 2. Materials and Methods

### 2.1. Data Source

FoodSwitch Hong Kong (FSHK) collected data on pre-packaged foods in Hong Kong annually between 2017–2019 using a standardized data collection procedure previously described in detail [29]. Data from the 2019 FSHK database were used in this study. In brief, the 2019 FSHK database includes information on all pre-packaged foods and beverages sold in five major supermarket chains including Wellcome and Park’nShop (selling local and imported products), AEON (selling products imported from Japan), City’Super (selling products imported internationally), as well as the retail company Marks & Spencer (selling private label products mainly from the UK). These stores sell a wide variety of products that are commonly consumed by Hong Kong citizens of different income levels. In particular, the two leading supermarket chains, Park’nShop and Wellcome account for a large proportion of the market share of the food-related retailing industry (62.5%) [30]. The largest store of each chain was selected for data collection, which was located in different geographical areas in Hong Kong, although all are considered affluent areas. Trained data collectors visited these stores between Jun–Aug 2019, and took photographs of the package, barcode, nutrition information panel (NIP), and ingredient list of each product. The photographs were then processed and stored by a data management center that was under the supervision of The George Institute for Global Health [31]. 

### 2.2. Definition of Free Sugar

This study used the WHO definition of free sugars, that is, all monosaccharides and disaccharides added to foods and beverages during processing, in addition to the naturally occurring sugars in honey, syrups, fruit juices, and fruit juice concentrates [7]. This definition does not include sugars that are naturally present in intact fruits (i.e., fructose) and vegetables, or those in milk or other unsweetened dairy products (i.e., lactose). It should be noted that according to the Food and Drugs (Composition and Labelling) Regulations in Hong Kong [32], sugar alcohols (e.g., sorbitol, mannitol) are not included in the definition of ‘sugars’ as they are neither monosaccharides or disaccharides.

### 2.3. Data Processing

The brand name, product name and package size as displayed on the package of each product were recorded in the FoodSwitch Hong Kong database. Nutrition information including the total sugar content of all pre-packaged products was obtained according to the NIP on the back-of-pack. If the total sugar content was shown per serving or per package, it was converted into g per 100 g or g per 100 mL.

### 2.4. Product Categorization and Exclusion

The George Institute for Global Health has developed a standard food categorization system [31], which classifies products into 20 major food groups and 81 minor food groups. There were 21,122 products recorded in the FoodSwitch Hong Kong database. ‘Special foods’ (*n* = 599) were excluded as they mainly consist of baby foods, which were not consumed by the majority of the population. Also, food items that were classified as ‘Unable to be categorized’ (*n* = 41), ‘Alcohol’ (*n* = 7), ‘Vitamin and minerals’ (*n* = 5), with a missing ingredients list (*n* = 20) or declaration of total sugar content (*n* = 1207), with multiple NIPs or a mixed assortment of food products (*n* = 39), and pack-size duplicate products (e.g., Brand A cola soft drink in 335 mL and 600 mL packages; *n* = 420) were excluded. 

### 2.5. Identification of Free Sugar Ingredients in Pre-Packaged Foods

FSIs were classified into 12 types (Table 1). The ingredient lists of all pre-packaged foods were transcribed from the FoodSwitch Hong Kong database. A glossary of all ingredient terms found in the database was created and 151 unique FSI-related ingredient terms were identified. FSI ingredients in pre-packaged foods were identified by searching ingredient list information for these terms, as previously described by Bernstein et al. [22]. Foods without FSI in their ingredient list were regarded as containing no free sugars during Step 2 of the estimation procedures, while those with FSI had their free sugar content estimated using other steps. 

### 2.6. Estimation of Free Sugar Content in Hong Kong Pre-Packaged Foods

Methodologies by Louie et al. [28] and Bernstein et al. [22] were adopted for estimation of free sugar content in pre-packaged foods. The modified method used in this study consisted of 8 steps: Steps 1–5 were considered as objective with higher confidence of estimation, while Steps 6–8 were regarded as subjective steps. The first author (L.Y.C.) applied the modified method to the 2019 FSHK dataset, with inputs from the last author (J.C.Y.L.) for difficult products. A detailed explanation and worked examples of each step are provided in Appendix A. 

### 2.7. Analysis of Naturally Occurring Lactose

High performance liquid chromatography (HPLC) is a common method to quantify the concentration of different types of sugar (e.g., glucose, fructose, lactose) in foods and beverages [33,34]. In Step 4, HPLC analysis was carried out to measure the intrinsic lactose content. A total of 105 products from 7 major categories, namely ‘Bread and bakery products’ (*n* = 47), ‘Confectionery’ (*n* = 24), ‘Dairy’ (*n* = 24), ‘Cereal and grain products’ (*n* = 4), ‘Non-alcoholic beverages’ (*n* = 3), ‘Snack foods’ (*n* = 2) and ‘Sauce, dressings, spreads and dips’ (*n* = 1), were selected for analysis. These products were composed of mainly dairy products and contain no fruits; as such, lactose is the only natural sugar present. An average lactose content was obtained from the triplicate measurements of each product. HPLC procedures are described in detail in Appendix B. 

### 2.8. Statistical Analysis

Data were analyzed using SPSS (version 26; IBM Corporation). Total sugar and free sugar content of pre-packaged foods were presented as means, standard deviations (SD), and quartiles (minimum, 25th, 50th, 75th, maximum) of total amounts in grams for 14 major food groups and 54 minor food groups (see Table S1 for the food groups examined). The proportion of pre-packaged products with FSI were presented for each major and minor food category. The contribution of free sugars as a percentage of total sugars of each food group was also presented.

## 3. Results

### 3.1. Distribution of Steps in the Systematic Methodology 

**Figure 1:**
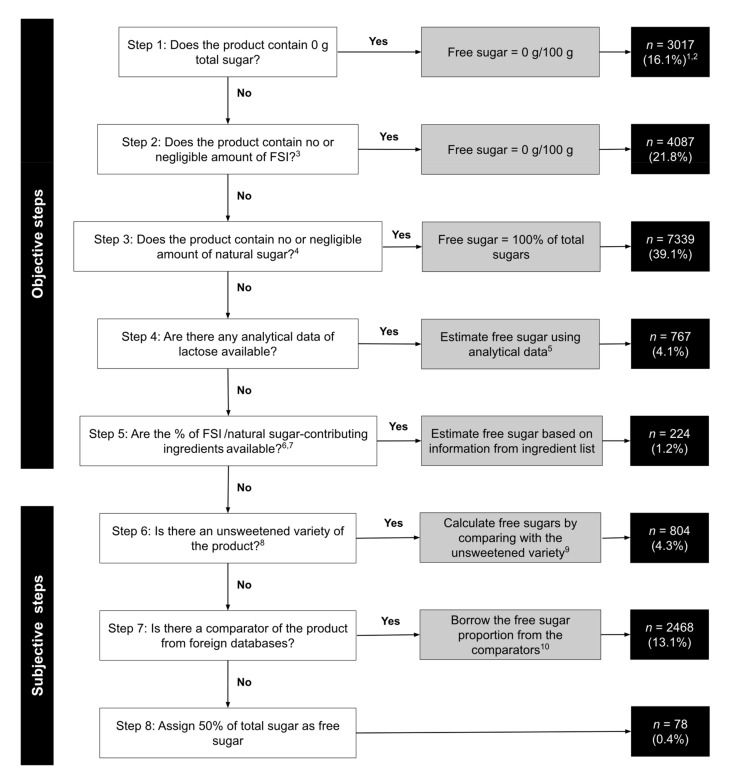
The decision algorithm for estimation of free sugars in pre-packaged products in the 2019 FoodSwitch Hong Kong database and the number of products in each step (total *n* = 18,784). Abbreviations: FSI = free sugar ingredient, NIP = Nutrition Information Panel. Footnote is available in Appendix C.

After applying the exclusion criteria, a total of 18,784 products from 14 major food groups and 54 minor food groups were included. Objective steps (Steps 1–5) were used to estimate the free sugar content of 82.2% of products and subjective steps (Steps 6–8) were used to estimate the free sugar content of the remaining 17.8% of products (Figure 1). Among all the steps, Step 3 (which checked for the presence of natural sugars in the products) was applied most extensively to estimate the free sugar content of almost 40% of products. 

### 3.2. Use of Free Sugar Ingredients

Among all audited pre-packaged foods, the majority of products (64.5%) contained at least one FSI (*n* = 12,118) (Table 2). Sucrose, either in solid or syrup forms, was the most commonly used FSI in the Hong Kong pre-packaged food supply and was identified in 54.7% of all included products. Furthermore, when only products with FSI were considered, 84.8% of them contained sucrose, followed by glucose (27.3%) and fruit juice (17.8%). Fructose and HFCS were only found in 1.7% and 2.3% of the audited products respectively. 

All major food groups contained products with FSI, even in food groups that were not often associated with free sugar such as ‘Fish and fish products’ (46.0%) and ‘Meat and meat products’ (73.8%) (Table 3). In 11 out of 16 major food groups, more than 50% of the products were found to contain FSI, in which ‘Sugars, honey and related products’ had the largest proportion of products with FSI (96.5%), followed by ‘Confectionery’ (93.5%) and ‘Bread and bakery products’ (91.2%). 

### 3.3. Median Total Sugar and Free Sugar Content

The median (IQR) free sugar content of all included products was 3.2 (0.0–17.2) g per 100 g or 100 mL (Table 3). ‘Sugars, honey and related products’ had the highest median free sugar level (80.0; 72.0–96.0), largely due to the high free sugar content contributed by its minor food categories, namely, ‘Sugar’ (99; 94.1–99.9), ‘Honey’ (78.1; 73.4–81.0) and ‘Syrup’ (73.6; 59.5–80.0). This category also included ‘Artificial sweeteners’ which had 0 g per 100 g free sugars. ‘Confectionery’ has the second-highest median free sugar (41.2; 25.4–58.9), since its two minor food groups ‘Chocolate and sweets’ (44.4; 32.8–59.2) and ‘Cough lollies’ (64.0; 0.0–76.0) contained high levels of free sugar (Table S2). The ‘Fruit’ category, which included a wide variety of fruit-related products such as fruit bars and fruit bites, had a large difference in the median total sugar (44.0; 17.0–62.7) and free sugar content (1.0; 0.0–16.6), implying that most of the sugars in these fruit products were naturally-occurring. Interestingly, ‘Fruit and vegetable juices’ had a higher median free sugar content (10.0; 8.3–11.5) than ‘Soft drinks’ (8.5; 6.0–10.6). It should also be noted that the distribution of free sugar level was highly skewed in several major food groups, with extreme outliers containing high levels of free sugars including in ‘Non-alcoholic beverages’, ‘Fruit and vegetables’, and ‘Dairy’ products (Figure S1).

### 3.4. Free Sugar as a Percentage of Total Sugar

Overall, free sugars constituted 65.8 ± 43.4% of the total sugars in all pre-packaged products, while the remaining proportion was coming from naturally occurring sugars (Table 3). Across all the major food groups, the free sugar percentage ranged from 1.8 ± 11.9% in ‘Edible oils and oil emulsions’ to 98.7 ± 11.2% in ‘Sugars, honey and related products’. In 8 of the 14 major food groups, the free sugar content made up over 70% of the total sugar content including in ‘Meat and meat products’ (80.2 ± 39.7%) and ‘Fish and fish products’ (73.9 ± 44.0%). However, in these categories, both the median free sugar content (g per 100 g) (0.9; 0.0–2.1 and 0.0; 0.0–2.8, respectively) and total sugar content (1.1; 0.3–2.2 and 0.6; 0.0–3.2, respectively) were not high and therefore free sugar constituted a great proportion of the total sugar content.

The free sugar content accounted for over 90% of total sugar in some minor food groups (Appendix C), including ‘Cough lollies’, ‘Jelly’, ‘Jam and Marmalades’, ‘Cordials’, ‘Electrolyte drinks’, ‘Energy drinks’, ‘Fruit and vegetable juices’, ‘Soft drinks’, and most of the minor food groups of ‘Sugars, honey and related products’. Notably, products that generally claimed to be ‘healthy’ such as cereal bars and fruit juices, also contained high levels of total sugars (26.0 ± 8.6 and 9.8 ± 3.8 g per 100 g or 100 mL) and a large proportion of total sugars as free sugars (76.4 ± 23.7% and 100.0 ± 0.0% respectively).

## 4. Discussion

This study revealed that around two-thirds of the 18,784 pre-packaged foods and beverages available for sale in Hong Kong were found to contain at least one FSI, showing the extensive use of free sugars in the Hong Kong pre-packaged food supply. Among all types of FSI, sucrose was the most popular, appearing in more than half (54.7%) of the analyzed products. Overall, the free sugar content comprised 65.8 ± 43.4% of the total sugar content. As expected, ‘Sugar, honey and related products’ had the highest median free sugar content (80.0; 72.0–96.0), and the largest contribution of free sugars to the total sugar content (98.7 ± 11.2%). 

The frequency of FSI found in Hong Kong pre-packaged foods (64.5%) was similar to that in Canada (63.5%) [22] but was higher than that in Slovenia (52.6%) [35], which might be due to the voluntary industrial commitments the Slovenian government has made to reduce the free sugar composition in foods. Surprisingly, food types that were not traditionally associated with free sugars (e.g., fish and meat products) also contained a considerable proportion of products with FSI, suggesting a wide usage of free sugars in food processing. Rather than acting as a sweetener, sometimes FSI is added for other purposes such as thickening, fermentation, and preservation of processed foods [36]. Nonetheless, the high prevalence of free sugars throughout the food supply suggests that consumers may be unaware of the amount of free sugars they are consuming, particularly as we have shown that free sugars are present in products that are not typically associated with free sugar, such as fish and meat products. While product reformulation for high sugar products (e.g., chocolate, sweets, cakes, and muffins) may be effective in achieving sugar reduction in some countries [37,38], reformulation may not be the most effective solution for reducing free sugar intakes in Hong Kong, where around 95% of food and beverage products are imported [39]. This suggests that in order to improve free sugar intakes across the population in Hong Kong, a greater focus needs to be applied on consumer knowledge and education to help consumers identify the main sources of free sugars as well as different FSI terms present in local products. While switching from high to low free sugar products should theoretically reduce free sugar consumption, experience in the UK regarding reducing the intake of salt, another negative nutrient of public health concern, suggests that the impact of product switching alone may be small [40]. A multi-strategy population-based approach is likely required to achieve meaningful reductions in the mean population free sugar intake. However, government and public health agencies should be careful when planning such interventions, such that health disparities between vulnerable populations and their healthier counterparts are not exacerbated [41]. 

Similar to pre-packaged foods in Brazil [21] and Canada [20,22], sucrose was the most frequently used FSI. While in the US, corn syrup was the most common sweetener [42], only 4.6% of the pre-packaged products in Hong Kong contained ‘corn syrup’. Corn is the most widely produced agricultural crop in the US, which accounts for almost half of the annual total agricultural revenue ($63.9 billion) [43]. The US government has taken some measures such as tariff-rate quotas to restrict imported sugar from entering the US market to support local sugar prices [44]. Therefore, it is more profitable to produce corn syrup than sugar in the US. Meanwhile, the two major importing countries of Hong Kong in terms of pre-packaged products—the U.K. and Japan—are active local cane sugar producers [45,46], which likely explains the larger proportion of products containing sucrose in Hong Kong as shown in this study.

Concerning the health impacts of different types of FSI, the derivative of corn syrup—HFCS—has been suggested to be associated with increased energy consumption that leads to obesity [47], high cholesterol [48,49] and metabolic disorders [50]—although whether such associations are unique to HFCS remain highly controversial [51,52]. In theory, unlike glucose, fructose from HFCS does not trigger insulin secretion, which might explain its relationship with weight gain, since insulin is a key energy-regulating hormone [2]. The chemical composition of sucrose (50% glucose and 50% fructose) is similar to that of HFCS (55% fructose and 45% glucose in beverages) and hence, they exert a similar metabolic effect after consumption [53]. The frequent use of sucrose in pre-packaged foods is therefore also of concern as it suggests the potential for an increase in the prevalence of obesity and consequential health problems [47] in Hong Kong. 

Fruit and vegetable juices are generally regarded as healthy alternatives to sugar-sweetened beverages (SSB), nevertheless, the median free sugar content in fruit and vegetable juices was found to be higher than that in soft drinks. Previously, sugars in vegetable juice or concentrates were not considered free sugars, but due to the broken cellular structure of vegetables in juices, there is no reason to consider these sugars any differently from that of sugars in fruit juices. As such, all sugars present in vegetable drinks are now considered free sugars [54,55]. This change means that previous epidemiology studies on free sugar may have under-estimated the intake of free sugars and its association with health outcomes. Given the rising popularity of fruit and vegetable juices in Hong Kong in recent years [56], it is likely that they contribute significantly to the total free sugar intake of the Hong Kong population, although there are no formal data confirming this assertion. Controversially, the Hong Kong Centre for Health Protection suggests consuming 180 mL pure fruit juice (without added sugar) as one serving of fruit to meet daily intake requirements [57], which is roughly equal to 60% of recommended free sugar intake. Although it remains unclear whether fruit juices can lead to similar metabolic effects as SSB [58], they are unlikely to be a healthy substitute for prevention of diseases such as type 2 diabetes given their high free sugar content [59]. 

Furthermore, carbonated drinks and fruit juices are popular in Hong Kong, representing 32% of total sugar intake [16]. In consideration of the high free sugar percentage (89.9%) of non-alcoholic drinks, economic measures such as taxation may be effective to discourage people from consuming these products and encourage reformulation [60,61]. Other countries such as the UK [62], Mexico [63], and Chile [64] have had some success in this area through introduction of a sugar tax. However, enormous opposition is expected from the beverage industry, which makes implementation of such policies very difficult [65]. Substitution of sugars with artificial sweeteners is also often used as a strategy for reducing dietary sugar intake. Studies in Europe [66,67] and in Hong Kong [68] have found that the use of low calorie and artificial sweeteners is increasingly more common, whereby manufacturers utilize them to achieve a lower sugar content in their products without affecting taste. The potential health effects of this increased prevalence of low calorie and artificial sweetener use in the population’s diet is largely unknown. While an early mice study suggests this may negatively affect the gut microbiome [69], a secondary analysis of a recent human trial suggest that regular consumption of artificial sweetener at typical high dietary dosages may have minimal impact on gut microbiome composition [70].

As shown in this study, a diverse range of terms are used to describe FSI on pre-packaged products sold in Hong Kong, which likely makes it difficult for consumers to identify whether a product contains free sugar, particularly if a product contains some less common FSI terms which may be difficult to recognize e.g., dextrose. Tierney et al. [71] reported that half of the 445 adult consumers surveyed in Northern Ireland could not classify ‘invert sugar’ and ‘isoglucose’ as added sugar ingredients. To tackle this issue, Ahuja et al. [72] has developed an improved framework for reporting ingredients in the US. They had identified some equivalent terms (e.g., synonyms) for a specific ingredient and assigned them with a preferred descriptor that can be easily recognized by consumers [72]. In Canada, it is now required to group all sugar-based ingredients as ‘Sugars’ in the list of ingredients [73], enabling consumers to identify unusual sources of free sugars and understand the proportion of free sugars in the products. Similar regulation policies may be needed in Hong Kong in order to standardize the names of different types of FSI on all pre-packaged items.

This study has adapted and applied a method to determine the free sugar content of products contained in a comprehensive database of pre-packaged products in Hong Kong. As such, this information could be combined with the dietary intake data from a population-based food consumption survey to assess the free sugar intake of Hong Kong citizens, which has already been carried out in other countries such as Australia and New Zealand [74,75,76]. These data would provide useful and accessible information to researchers and policymakers to implement public health interventions and policies (e.g., free sugar labeling) that aim to reduce free sugar consumption. The data on FSI use and estimated free sugar content can also serve as baseline data to monitor the progress of free sugar reduction initiatives in the future [37]. Nonetheless, the frequent introduction of new products, short product lives, and changes to product formulation make it challenging to keep a database of nutrition information of pre-packaged food up to date [77,78]. Approaches such as webscraping of supermarket websites to automate data collection, as described by Harrington et al. [78], is a promising solution to this problem. The objective steps in our approach may also be automated into a decision-tree-based computer algorithm [79,80] to reduce the burden of regular updates to the database.

Our study has several notable strengths. First, we obtained analytical data on sugar content using HPLC (which were often unavailable in other studies that utilized a similar methodology [22,37]), which enhanced the objectivity of this method. To date, studies analyzing sugar content in foods and beverages in Hong Kong have had a small sample size, limited coverage of food categories, and have not assessed the free sugar content [81,82]. Our study used an extensive database covering supermarket chains that make up the majority of the market share, providing free sugar data of a comprehensive range of pre-packaged products sold in Hong Kong. Moreover, the free sugar content of over 80% of the 18,784 products in the database were estimated objectively, minimizing subjective biases.

We caution readers to some limitations of our study. First, the nutritional information of the ‘as consumed’ form is not mandatory in Hong Kong; thus, the free sugar content of all products was estimated based on the foods’ ‘in the form ready for sale’ [32], which does not reflect the true free sugar value for products that are purchased as convenience mixes (e.g., dessert mixes) and require further preparation (e.g., reconstitution of milk powder) before consumption. Although the number of such products are small (<3%) and were mostly found in ‘non-alcoholic beverages’, ‘cereals and cereal grain products’ and ‘convenience foods’, this introduced significant outliers (Figure S1). Second, the rounding rules allowed on nutrition labels limited the precision of the estimated values. Food labeling regulations of Hong Kong [32] allows a 0.5 g per 100 g or 100 mL tolerance for pre-packaged products that are claimed to be sugar-free (i.e., listed as 0.0 g per 100 g or 100 mL on their nutrition label). Consequently, 345 (11.4% of all foods in Step 1) foods that claimed to have 0 g total sugars were found to have at least one FSI in their ingredient lists. Similarly, the borrowed data from the USDA database includes added sugar values from Nutrition Facts labels, which allows label rounding [83,84,85]; hence, the results may vary from those obtained by analytical measurement. Third, limitations may also arise during the identification of FSIs. The ingredient lists of some products showed sweetened composite ingredients (e.g., condensed milk, chocolate paste) instead of the FSI itself. Hence, some of the products (e.g., desserts) were mistaken as free of FSI and assigned a 0 g free sugar value in Step 2. However, this only occurred for 19 products (0.5% of all foods in Step 2) and therefore it is unlikely to have caused a substantial change in the free sugar content. Lastly, although this method tried to maximize its objectivity by adopting chemical analysis, there were subjective decisions involved when choosing steps for analysis, which may affect the repeatability of the method, especially among the subjective steps [28]. Although not formally explored in the current study, our group previously demonstrated good inter-rater repeatability of a similar algorithm [28], supporting the utility of this method as a standardized approach for estimation of free sugars in large-scale studies.

## 5. Conclusions

Free sugars are highly prevalent in the pre-packaged food supply in Hong Kong, with a wide distribution of free sugars in over two-thirds of the analyzed food items, including foods that are not typically associated with being high in sugar such as fish and meat products. Further studies are needed to assess the population intake of free sugars in Hong Kong to inform proper policy decisions on free sugar reduction.

## Figures and Tables

**Table 1:** Types of FSI and examples of terms for each FSI identified from the ingredient lists of 18,784 pre-packaged products.

Type of FSI	Examples of Common Terms ^a^
***Sucrose (sugar & syrup)*** ^b^	Beet sugar, cane sugar, caster sugar, demerara sugar, granulated sugar muscovado sugar, sucrose, sugar, turbinado sugar, sugar cane juice, caramel, brown sugar syrup, cane sugar syrup
Glucose	Corn maltodextrin, D-glucose powder, dehydrated glucose syrup, dextrose, dextrose monohydrate, isomerized glucose syrup, rice syrup
Corn Syrup	Corn glucose syrup, corn syrup, fructose dextrose syrup, fructose glucose, high maltose corn syrup
Fruit juice	Fruit juice concentrate, fruit juice powder, concentrated fruit juice, fruit nectar, concentrated grape must, dried fruit juice
High-fructose corn syrup	Fructose corn syrup, high fructose corn syrup, high fructose glucose syrup, high fructose syrup, isomerized syrup
Molasses	Beet molasses, blackstrap molasses, cane molasses, black treacle
Honey	Honey, honey syrup, brown honey, oak honey, raw honey
Other syrups	Barley malt extract, cider syrup, coconut syrup, malt syrup, lactose solution, invert liquid sugar
Fructose	Crystalline fructose, fructose, fructose syrup, fruit sugar
Other sugars	Coconut sugar, dried barley malt extract, maltose, oak sugar, palm sugar, invert sugar
Maple syrup	Maple sugar, maple syrup
Agave	Agave nectar, agave powder, agave syrup

^a^ 151 unique terms of FSI were found, without considering differences in spellings, place of origins (e.g., European sunflower honey), fruit flavors, degree of purity (e.g., 100% pure honey), ‘natural’ or ‘organic’ variations (e.g., natural/organic honey). ^b^ Caramel used as a coloring agent as specified by the ingredient list was not considered an FSI. Source: Adapted from Bernstein et al. [22].

**Table 2:** Prevalence of use of free sugar ingredients (FSI) among products in the 2019 FoodSwitch Hong Kong database.

Type of FSI ^a^	*n*	Prevalence (%) in Products that Contain FSI (*n* = 12,118) ^b^	Prevalence (%) in All Products (*n* = 18,784) ^b^
Sucrose (sugar & syrup)	10,282	84.8	54.7
Glucose	3305	27.3	17.6
Fruit juice	2154	17.8	11.5
Other sugars	995	8.2	5.3
Other syrups	909	7.5	4.8
Corn syrup	857	7.1	4.6
Honey	727	6.0	3.9
High-fructose corn syrup	425	3.5	2.3
Fructose	311	2.6	1.7
Molasses	171	1.4	0.9
Maple syrup	53	0.4	0.3
Agave	39	0.3	0.2

^a^ FSIs were shown by descending order of use. ^b^ The combined percentages of all FSIs would be greater than 100% as some products contain more than one FSI.

**Table 3:** Total and free sugar contents (g/100 g or g/100 mL) and mean free sugars as a percentage of total sugars (%) of products in the 2019 FoodSwitch Hong Kong database.

Major Food Groups ^a^	Total *n*	*n* (%) Containing FSI(s)	Total Sugar Content (g/100 g or g/100 mL)						Free Sugar Content (g/100 g or g/100 mL)						Free Sugar as Percent of Total Sugar ± SD (%) ^b^
			Mean ± SD	Min	25th	50th	75th	Max	Mean ± SD	Min	25th	50th	75th	Max	
Bread and bakery products	1719	1567 (91.2)	22.7 ± 15.1	0.0	10.0	23.2	33.0	90.7	20.8 ± 15.0	0.0	7.0	21.6	31.1	90.7	80.6 ± 34.5
Cereal and grain products	2673	1152 (43.1)	6.8 ± 10.5	0.0	0.5	2.8	7.0	86.5	4.5 ± 8.8	0.0	0.0	0.0	4.5	68.7	46.2 ± 47.5
Confectionery	1624	1519 (93.5)	46.4 ± 26.0	0.0	29.0	48.3	61.1	100.0	43.1 ± 26.4	0.0	25.4	41.2	58.9	100.0	89.5 ± 19.6
Convenience foods	1074	851(79.2)	4.7 ± 6.9	0.0	1.1	2.3	4.9	57.3	3.2 ± 6.0	0.0	0.0	0.8	2.9	45.2	50.8 ± 42.3
Dairy	1573	919 (58.4)	9.8 ± 10.8	0.0	2.8	6.9	13.4	96.6	6.1 ± 9.4	0.0	0.0	3.5	9.0	96.6	45.9 ± 39.5
Edible oils and oil emulsions	458	6 (1.3)	0.2 ± 1.3	0.0	0.0	0.0	0.0	26.1	0.6 ± 1.1	0.0	0.0	0.0	0.0	23.2	1.8 ± 11.9
Eggs	71	3 (4.2)	0.6 ± 2.8	0.0	0.0	0.0	0.3	20.9	0.5 ± 2.8	0.0	0.0	0.0	0.0	20.9	11.5 ± 32.6
Fish and fish products	605	278 (46.0)	2.1 ± 3.9	0.0	0.0	0.6	3.2	60.2	1.9 ± 3.9	0.0	0.0	0.0	2.8	60.2	73.9 ± 44.0
Fruits and vegetables	2406	893 (37.1)	15.8 ± 21.8	0.0	1.0	4.7	20.4	89.4	8.2± 16.9	0.0	0.0	0.0	5.3	86.0	33.3 ± 43.7
Meat and meat products	660	487 (73.8)	2.6 ± 5.5	0.0	0.3	1.1	2.2	49.6	2.4 ± 5.5	0.0	0.0	0.9	2.1	49.6	80.2 ± 39.7
Non-alcoholic beverages	2246	1498 (66.7)	12.5 ± 17.7	0.0	1.0	8.1	11.5	100.0	11.9 ± 17.3	0.0	0.0	8.0	11.4	100.0	89.9 ± 28.2
Sauce, dressings, spreads and dips	2207	1695 (76.8)	12.4 ± 14.2	0.0	2.6	5.7	18.0	80.7	10.9 ± 14.1	0.0	0.0	4.8	16.7	80.7	71.9 ± 39.9
Snack foods	981	780 (79.5)	10.3 ± 13.8	0.0	1.8	3.8	11.4	68.6	8.8 ± 12.9	0.0	0.3	3.4	9.7	66.0	82.5 ± 37.0
Sugars, honey and related products	487	470 (96.5)	77.7 ± 21.9	0.0	72.0	80.0	96.0	100.0	77.4± 22.5	0.0	72.0	80.0	96.0	100.0	98.7 ± 11.2
Total	18,784	12,118 (64.5)	15.8 ± 22.0	0.0	1.2	5.7	21.4	100.0	13.3 ± 21.2	0.0	0.0	3.2	17.2	100.0	65.8 ± 43.4

^a^ Six food groups were excluded from the analysis, including ‘alcohol’, ‘vitamins and supplements’, ‘unable to be categorized’, ‘special foods’, ‘fast foods and takeaways’ and ‘generic/non-branded items’. ^b^ Calculated as (free sugar/total sugar content) × 100%. Abbreviation: FSI = free sugar ingredient.

## Data Availability

Due to the proprietary nature of the data, interested parties should contact Fraser Taylor (ftaylor@georgeinstitute.org.au), Managing Director of the Food Policy Division, George Institute for Global Health, Australia and Jimmy Louie (jimmyl@hku.hk) of the School of Biological Sciences, The University of Hong Kong, for permission to access the dataset described in this manuscript (fees may apply).

## Supplementary Materials

The following are available online at https://www.mdpi.com/article/10.3390/nu13103404/s1. Table S1: The major and minor food groups examined in this study; Table S2: Total and free sugar contents (g/100 g or g/100 mL) and mean free sugars as a percentage of total sugars (%) of pre-packaged products in the 2019 *FoodSwitch* Hong Kong Database (total *n* = 18,784); Figure S1: Median and interquartile range of free sugar content (g/100 g or 100 mL) by major food groups, in descending order of free sugar content. Asterisks (*) represent outliers.

## Appendix A. Detailed Explanation and Worked Examples of Each Step

**Step 1: Assign products that contain 0 g total sugar as declared on the FoodSwitch HK database as free sugar value = 0 g/100 g.**

Explanation: Products containing 0 g of total sugars should contain 0 g free sugars.

Example:

Product: Voortman Sugar Free Oatmeal Cookies

Total sugar content (per 100 g): 0 g

Free sugar content (per 100 g): 0 g

**Step 2: Assign products that contain no or a negligible amount (<0.5 g per 100 g or 100 mL) of free sugar ingredient (FSI) as free sugar = 0 g/100 g.**

Explanation: since free sugars can only be provided by FSI(s) by definition, products with little or no FSI should contain 0 g of free sugar.

Example 1: Products with no FSI

Product: Carr’s Cream Crackers

Ingredient list: wheat flour, vegetable oil, palm, salt, yeast, raising agent, sodium bicarbonate

Total sugar content (per 100 g): 1.4 g

Free sugar content (per 100 g): 0 g

Example 2: Products with a negligible amount of FSI

Product: Bagels Forever Plain

Ingredient list: enriched high gluten flour, wheat flour, malted barley flour, niacin, reduced iron, thiamin mononitrate, riboflavin, folic acid, water, malt, salt, **sugar**, cornmeal, yeast, enzyme

Total sugar content (per 100 g): 4.7 g

Free sugar content (per 100 g): 0 g

**Step 3: Assign products that contain no or a negligible amount of intrinsic sugars as free sugar = 100% of total sugar.**

Explanation: Since total sugar = natural/intrinsic sugar + free sugar, if a product contains very little or no natural/intrinsic sugar, then total sugar should be the same as free sugar.

Example 1: Products that belonged to the ‘100% free sugar food groups’

Product: Coca-Cola Coke

Total sugar content (per 100 g): 11.2 g

Free sugar content (per 100 g): 11.2 g

*Example 2:* Products that contained no or minimal amounts of fruits and dairy products

Product: Admiral Ice Cream Cones

Ingredient list: wheat flour, sugar, palm oil, soya lecithin, caramel, e150

Total sugar content (per 100 g): 39 g

Free sugar content (per 100 g): 39 g

*Example 3:* Products that contained no or minimal amounts of fruits and dairy products

Product: Dare Breton Wheat Crackers Original

Ingredient list: wheat flour, vegetable oil shortening, coconut oil, canola oil, wheat germ, whole wheat flour, sugar, salt, whey (milk), raising agent, e503, e500, hydrolyzed soy protein, natural flavour

Total sugar content (per 100 g): 6.4 g

Free sugar content (per 100 g): 6.4 g

**Step 4: Use of analytical data of lactose for products with dairy ingredients.**

Explanation: Similar to step 3, since total sugar = natural/intrinsic sugar + free sugar, for products that contained lactose as the only intrinsic sugar, free sugar = total sugar − lactose.

Example:

Product: Cadbury Dairy Milk Chocolate

Ingredient list: full cream milk, sugar, cocoa butter, cocoa mass, milk solids, emulsifier, soy lecithin, e476, flavour

Analytical data for natural lactose content: 9.8 g

Total sugar content (per 100 g): 57.1 g

Free sugar content (per 100 g): 57.1 − 9.8 g = 47.3 g

**Step 5: Calculate free sugar content for products with a known content of FSI or natural sugar-contributing ingredients.**

Explanation: Similar to step 3, since total sugar = natural/intrinsic sugar + free sugar, free sugar content could be calculated if the content of the natural/intrinsic sugar-containing ingredient is known; and vice versa.

Example 1: Products with a known content of FSI

Product: Oriental Layer Cake Banana Flavour

Ingredient list: egg, 33%, wheat flour, 32%, icing sugar, 25.29%, banana puree, 2%, shortening, palm oil, 6%, milk powder, 1%, raising agent, sodium bicarbonate, 0.6%, banana flavour, 0.11%

Free sugar concentration of icing sugar: 100 g/100 g

Total sugar content (per 100 g): 28.3 g

Free sugar content (per 100 g):

$$\sum_{i}^{j}{\mathrm{FSI}}_{i}{\left(\%)\times [\mathrm{free}\mathrm{sugar}\mathrm{concentration}\right]}_{i}$$

= 25.3% × (100 g/100 g)

= 25.3 g

Example 2: Products with a known content of natural sugar-contributing ingredients

Product: Kellogg’s Sultana Bran

Ingredient list: whole wheat, 37%, sultanas, 26%, wheat bran, 25%, sugar, barley malt extract, salt, humectant, glycerol, mineral, iron, zinc oxide, vitamins, niacin, riboflavin, vitamin b6, thiamin, folate

Natural sugar concentration of sultana (per 100 g): 59 g

Total sugar content (per 100 g): 22.7 g

Free sugar content (per 100 g):

$$\mathrm{Total}\mathrm{sugar}-(\sum_{i}^{j}{\mathrm{Natural}\mathrm{sugar}\mathrm{ingredient}}_{i}{\left(\%)\times [\mathrm{natural}\mathrm{sugar}\mathrm{concentration}\right]}_{i})$$

= 22.7−(26% × 59 g)

= 7.4 g

**Step 6: Calculate based on comparison with an unsweetened variety.**

Explanation: This step was adopted from Louie et al.

Example:

The unsweetened product

Product: Bulla Greek Style Gluten Free Natural Yoghurt

Ingredient list: fresh skim milk, fresh cream, milk solids, live yoghurt culture

Total sugar content (per 100 g): 8 g

Free sugar content (per 100 g): 0 g (obtained in Step 2 since it did not contain any FSI)

The sweetened product

Product: Bulla Family Dairy Plain Yoghurt

Ingredient list: fresh skim milk, milk solids, liquid sugar, sugar, water, thickener, e1442, from tapioca and maize, fresh cream, cultures

Total sugar content (per 100 g): 10.3 g

Free sugar content (per 100 g): $\frac{{100\times (S}_{\mathrm{us}}{-S}_{\mathrm{total}})}{{(S}_{\mathrm{us}}-100)}=\frac{100\times (8\;g-10.3\;g)}{\left(8\;g-100\right)}$= 2.5 g

**Step 7: Borrow free sugar values from similar products from foreign food composition databases (e.g., USDA added sugar table** [84]**; AUSNUT2011–2013** [85]**).**

Explanation: An assumption that a similar product from a foreign database would have a similar formulation as the target product is made here.

Example:

Borrowed product

Description: *Jelly, sugar sweetened, all flavours, prepared, added berries*

Total sugar content (per 100 g): 10.9 g

Free sugar content (per 100 g): 9.3 g

Target product

Product: M&S Raspberry Jelly

Ingredient list: water, raspberries 18%, sugar, gelling agent, carrageenan, locust bean gum, e508, acidity regulator, citric acid, e331, flavouring, colour, anthocyanin, from black carrot

Total sugar content (per 100 g): 11.5 g

Free sugar content (per 100 g):

Totalsugarsoftargetproduct×borrowedproportionoffree(added)sugar

$=11.5\;g\times \left(\frac{9.3\;g}{10.9\;g}\right)$

= 9.8 g

**Step 8: Assign 50% of total sugar as free sugar.**

Explanation: A crude assumption that half of the total sugar is from free sugar is made here; given products with negligible or low free sugar content should have had their free sugar content estimated using an earlier step.

Example:

Product: Malay house durian mochi

Ingredient list: durian puree, full cream milk, glutinous rice flour, rice flour, wheat starch, margarine, icing sugar, potato starch

Total sugar content (per 100 g): 9.1 g

Free sugar content (per 100 g): 9.1 g × 50% = 4.6 g

## Appendix B. HPLC Procedures

### Appendix B.1. Sample Preparation

A total of 1 g of the sample was dissolved in a 10 mL 1:1 mixture of water and ethanol. For solid food samples, grinding and blending were necessary to maximize the extraction efficiency. The sample was then kept at 80 °C for 30 min and vortexed every 10 min. Later, the mixture was centrifuged at 5000× *g* for 40 min at room temperature. Then 1 mL of the supernatant was collected and centrifuged at 18,000× *g* for 20 min. Finally, the supernatants of the samples were filtered with a 0.22 μm polytetrafluoroethylene (PTFE) filter before transferring to an autosampler vial.

### Appendix B.2. Conditions for Analysis

The standards and samples were analyzed by a Luna Omega 3 μm SUGAR 100Å LC column (150 × 4.6 mm). The column was connected to an isocratic HPLC system and an RID detector. Acetonitrile (80%) was mixed with Milli Q water (20%) as the mobile phase. The flow rate was set at 0.8 mL/min and the column temperature was maintained at 50 °C. An injection volume of 3 μL was used to reach an optimal separation of peaks.

## Appendix C

Further Explanation of Figure 1

1. Number and proportion (%) of products calculated in each step. All products were calculated based on the total sugar content of their ‘ready for sale’ form as shown on their NIPs. The combined percentages of all steps were not equal to 100% due to rounding.
2. Step 1 included 345 items that claimed to have 0 g total sugars but were found to have at least one FSI in their ingredient lists.
3. Ingredient list of each product was searched for the identification of FSI. FSI was defined as all monosaccharides and disaccharides added to foods and beverages during processing, in addition to the naturally occurring sugars in honey, syrups, fruit juices, and fruit juice concentrates [7].
4. Include all products from the ‘100% free sugars groups’ (energy drinks, electrolyte drinks, soft drinks, fruit and vegetable juices, and cordials), in addition to those that were composed of no or minimal amounts of fruit and dairy products.
5. If the product contains lactose as the only naturally occurring sugar, the free sugar content was calculated as total sugar–lactose.
6. If the percentage of all FSI(s) in the product were explicitly stated in the ingredient list, free sugar was determined by $\sum_{i}^{j}{\mathrm{FSI}}_{i}{\left(\%)\times [\mathrm{free}\mathrm{sugar}\mathrm{concentration}\right]}_{i}$*,* where FSI*_i_* is the ith free sugar ingredient and *j* is the total number of FSI in the ingredient list.
7. If the proportion of all-natural sugar-contributing agents and their corresponding natural sugar concentrations were known, free sugar could be calculated by $\mathrm{Total}\mathrm{sugar}-\left(\sum_{i}^{j}{\mathrm{Natural}\mathrm{sugar}\mathrm{ingredient}}_{i}\left(\%\right)\times {\left[\mathrm{natural}\mathrm{sugar}\mathrm{concentration}\right]}_{i}\right)$, where Natural sugar ingredient*_i_* is the *i*th ingredient that contributes naturally occurring sugars and *j* is the total number of natural sugar ingredients in the ingredient list.
8. Products that had been given a free sugar value of 0 g in Step 1 or 2.
9. Free sugar content was calculated by $\frac{{100\times (S}_{\mathrm{us}}{-S}_{\mathrm{total}})}{{(S}_{\mathrm{us}}-100)}$, where $S_{\mathrm{us}}$ is the total sugar content (per 100 g) of the unsweetened counterpart of the product and $S_{\mathrm{total}}$ is the total sugar content of the sweetened product.
10. The borrowed proportion was substituted into: total sugars of target product × borrowed proportion of free(added) sugar.
