# Supplementary material for: Total and Free Sugar Levels and Main Types of Sugars Used in 18,784 Local and Imported Pre-Packaged Foods and Beverages Sold in Hong Kong
Source: Nutrients. 2021 Sep 27;13(10):3404. doi: 10.3390/nu13103404 (PMC8540970; doi:10.3390/nu13103404)
Supplement: Supplementary file 1 [file nutrients-13-03404-s001.zip › nutrients-1378611-supplementary/FigureS1.pdf]

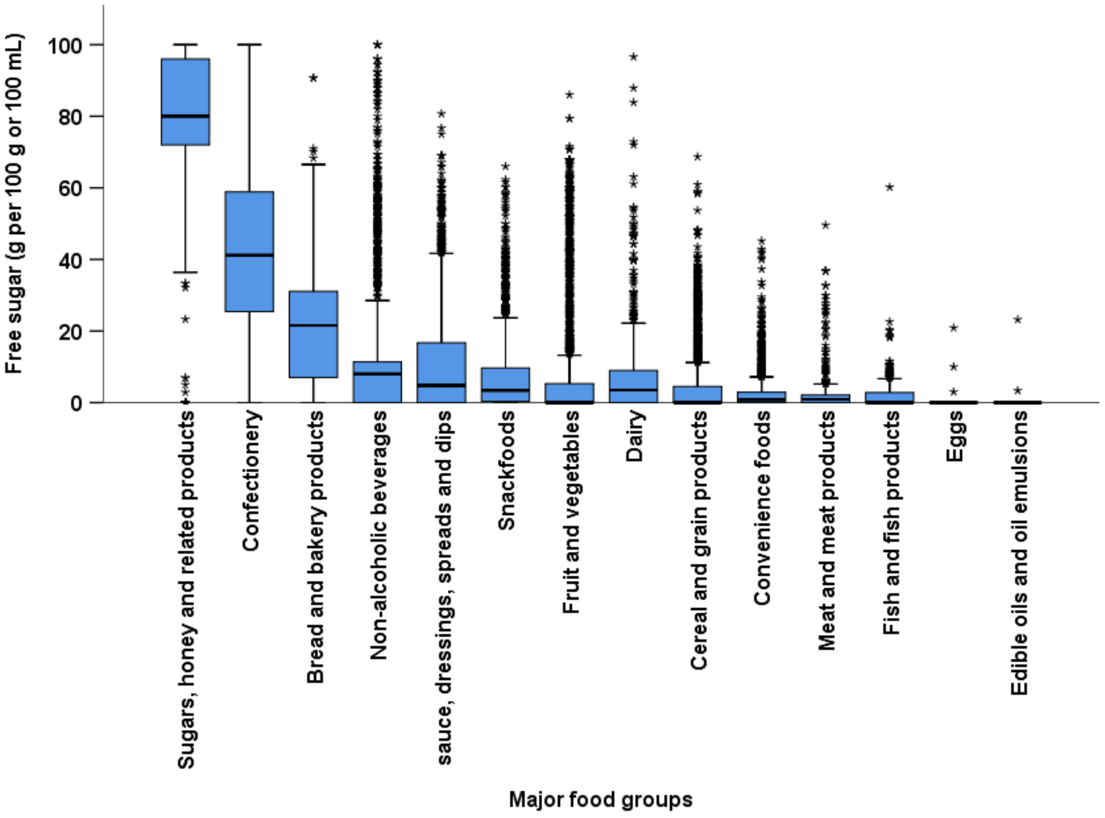

Figure S1. Median and interquartile range of free sugar content (g per 100 g or 100 mL) by major food groups, in descending order of free sugar content. Asterisks (*) represent outliers.
