# Supplementary material for: Total and Free Sugar Levels and Main Types of Sugars Used in 18,784 Local and Imported Pre-Packaged Foods and Beverages Sold in Hong Kong
Source: Nutrients. 2021 Sep 27;13(10):3404. doi: 10.3390/nu13103404 (PMC8540970; doi:10.3390/nu13103404)
Supplement: Supplementary file 1 [file nutrients-13-03404-s001.zip › nutrients-1378611-supplementary/TableS1_final.pdf]

Table S1 – The major and minor food groups examined in this study

Major food group	Minor food group
Bread and bakery products	- Biscuits
	- Bread
	- Cakes, muffins and pastries
Cereal and grain products	- Breakfast cereals
	- Cereal and nut-based bars
	- Couscous
	- Noodles
	- Pasta
	- Rice
	- Other cereal and grain products
Confectionery	- Chocolate and sweets
	- Jelly
	- Chewing gum
	- Cough lollies
Convenience foods	- Pizza
	- Pre-prepared salads and sandwiches
	- Ready meals
	- Soup
	- Meal kits
	- Other frozen foods not otherwise specified
Dairy	- Cheese
	- Cream
	- Desserts
	- Ice cream and edible ices
	- Milk
	- Yoghurt and yoghurt drinks
Edible oils and oil emulsions	- Cooking oils
	- Edible oils
	- Cooking oil spray
	- Coconut oil
Eggs	
Fish and fish products	
Fruit and vegetables	- Fruit
	- Herbs and spices
	- Jam and marmalades
	- Nuts and seeds
	- Vegetables
Meat and meat products	- Meat alternatives
	- Processed meat
Non-alcoholic beverages	- Coffee and tea

**Major food group**	**Minor food group**
	- Cordials
	- Electrolyte drinks
	- Energy drinks
	- Fruit and vegetable juices
	- Soft drinks
	- Waters
	- Beverage mixes
Sauces, dressings, spreads and dips	- Mayonnaise and salad dressings
	- Sauces
	- Spreads and dips
Snack foods	
Sugars, honey and related products	- Honey
	- Dessert toppings
	- Syrup
	- Dessert additions
	- Condensed caramel
	- Sugar
	- Sweeteners
