# Supplementary material for: Total and Free Sugar Levels and Main Types of Sugars Used in 18,784 Local and Imported Pre-Packaged Foods and Beverages Sold in Hong Kong
Source: Nutrients. 2021 Sep 27;13(10):3404. doi: 10.3390/nu13103404 (PMC8540970; doi:10.3390/nu13103404)
Supplement: Supplementary file 1 [file nutrients-13-03404-s001.zip › nutrients-1378611-supplementary/TableS2.pdf]

Table S2. Total and free sugar contents (g/100 g or g/100 mL) and mean free sugars as a percentage of total sugars (%) of pre-packaged products in the 2019 *FoodSwitch* Hong Kong Database (total *n* = 18,784).

Major and minor food groups	Total *n*	*n* (%) containing FSI(s)	Total sugar content (g/100 g or g/100 mL)						Free sugar content (g/100 g or g/100 mL)						Free sugar as percent of total sugar ± SD (%)[1]
			Mean ± SD	Min	25th	50th	75th	Max	Mean ± SD	Min	25th	50th	75th	Max	
**Bread and bakery products**	1719	1567 (91.2)	22.7 ± 15.1	0.0	10.0	23.2	33.0	90.7	20.8 ± 15.0	0.0	7.0	21.6	31.1	90.7	80.6 ± 34.5
Biscuits	1040	951 (91.4)	23.4 ± 14.2	0.0	13.5	24.7	33.7	70.2	21.7 ± 13.6	0.0	12.6	22.5	32.0	70.2	88.0 ± 25.6
Bread	180	147 (81.7)	7.2 ± 7.4	0.0	2.6	4.7	10.3	39.0	3.3 ± 7.0	0.0	0.0	0.0	2.9	39.0	22.5 ± 38.8
Cakes, muffins and pastries	499	469 (94.0)	27.0 ± 15.4	0.0	17.2	26.7	35.3	90.7	25.1 ± 15.7	0.0	14.9	25.3	33.6	90.7	86.2 ± 29.0
**Cereal and grain products**	2673	1152 (43.1)	6.8 ± 10.5	0.0	0.5	2.8	7.0	86.5	4.5 ± 8.8	0.0	0.0	0.0	4.5	68.7	46.2 ± 47.5
Breakfast cereals	463	323 (69.8)	16.1 ± 11.4	0.0	6.0	16.5	23.9	63.6	11.7 ± 11.2	0.0	0.0	9.9	20.0	48.0	58.1 ± 42.5
Cereal and nut -based bars	117	112 (95.7)	26.0 ± 8.6	9.3	19.9	26.0	33.1	47.0	19.4 ± 8.7	0.0	13.1	18.5	26.3	39.3	76.4 ± 23.7
Couscous	12	3 (25.0)	3.2 ± 2.3	0.3	1.9	3.0	4.0	9.0	0.7 ± 1.7	0.0	0.0	0.0	0.5	6.0	10.6 ± 21.2
Noodles	1004	596 (59.4)	4.1 ± 7.6	0.0	0.6	2.4	4.5	86.5	3.3 ± 6.8	0.0	0.0	1.4	4.2	60.9	70.1 ± 45.8
Other cereal and grain products (e.g., bread mixes, flour)	450	61 (13.6)	4.3 ± 10.5	0.0	0.0	0.9	3.4	77.3	1.9 ± 8.0	0.0	0.0	0.0	0.0	68.7	15.4 ± 35.3
Pasta	389	49 (12.6)	3.2 ± 2.1	0.0	2.0	3.4	3.7	14.0	0.3 ± 1.1	0.0	0.0	0.0	0.0	9.6	6.2 ± 21.3
Rice	239	8 (3.3)	1,4 ± 6.5	0.0	0.0	0.0	0.5	67.7	0.2 ± 1.3	0.0	0.0	0.0	0.0	13.2	6.9 ± 25.5
**Confectionery**	1624	1519 (93.5)	46.4 ± 26.0	0.0	29.0	48.3	61.1	100.0	43.1 ± 26.4	0.0	25.4	41.2	58.9	100.0	89.5 ± 19.6
Chocolate and sweets	1318	1254 (95.1)	51.0 ± 22.3	0.0	39.1	50.9	62.2	100.0	47.1 ± 23.5	0.0	32.8	44.4	59.2	100.0	88.7 ± 18.5
Chewing gum	55	29 (52.7)	11.5 ± 28.5	0.0	0.0	0.0	0.0	96.7	11.5 ± 28.5	0.0	0.0	0.0	0.0	96.7	66.7 ± 49.2
Cough lollies	58	52 (89.7)	45.2 ± 38.3	0.0	0.0	64.0	76.0	98.9	45.2 ± 38.3	0.0	0.0	64.0	76.0	98.9	97.4 ± 16.0
Jelly	193	184 (95.3)	25.0 ± 26.0	0.0	11.2	16.3	22.6	94.8	24.4 ± 26.3	0.0	10.4	14.4	22.0	94.8	94.9 ± 22.4
**Convenience foods**	1074	851(79.2)	4.7 ± 6.9	0.0	1.1	2.3	4.9	57.3	3.2 ± 6.0	0.0	0.0	0.8	2.9	45.2	50.8 ± 42.3
Meal kits	54	41 (75.9)	9.3 ± 9.4	0.0	2.0	6.6	14.7	42.9	7.5 ± 9.1	0.0	0.0	5.3	10.6	42.9	78.4 ± 40.1
Other frozen foods not otherwise specified	2	2 (100.0)	22.1 ± 16.5	10.4	10.4	22.1	/	33.8	13.7 ± 4.6	10.4	10.4	13.7	/	16.9	75.0 ± 35.4
Pizza	36	34 (94.4)	6.9 ± 8.1	1.3	2.5	3.6	5.4	27.9	5.0 ± 8.1	0.0	0.7	1.5	3.3	26.1	49.3 ± 31.5
Pre-prepared salads and sandwiches	69	65 (94.2)	2.6 ± 1.9	0.1	1.5	2.1	3.6	8.2	1.1 ± 1.1	0.0	0.2	0.9	1.3	5.5	40.9 ± 31.9
Ready meals	629	540 (85.9)	4.4 ± 6.1	0.0	1.1	2.3	4.8	40.4	3.3 ± 5.6	0.0	0.0	1.0	3.6	40.4	59.9 ± 43.2
Soup	284	169 (59.5)	4.3 ± 8.0	0.0	0.8	1.7	3.6	57.3	2.2 ± 6.1	0.0	0.0	0.0	1.3	45.2	27.5 ± 33.2
**Dairy**	1573	919 (58.4)	9.8 ± 10.8	0.0	2.8	6.9	13.4	96.6	6.1 ± 9.4	0.0	0.0	3.5	9.0	96.6	45.9 ± 39.5
Cheese	313	17 (5.4)	1.7 ± 2.6	0.0	0.0	0.5	2.3	14.7	0.2 ± 1.2	0.0	0.0	0.0	0.0	12.5	2.0 ± 11.2
Cream	35	12 (34.3)	4.8 ± 3.1	1.6	3.0	3.9	5.7	15.0	1.7 ± 3.5	0.0	0.0	0.0	1.6	11.3	21.3 ± 38.6

Major and minor food groups	Total *n*	*n* (%) containing FSI(s)	Total sugar content (g/100 g or g/100 mL)						Free sugar content (g/100 g or g/100 mL)						Free sugar as percent of total sugar ± SD (%)[1]
			Mean ± SD	Min	25th	50th	75th	Max	Mean ± SD	Min	25th	50th	75th	Max	
Desserts	177	145 (81.9)	16.0 ± 15.6	0.0	8.2	11.1	18.5	83.9	13.5 ± 15.2	0.0	4.9	8.9	15.8	83.9	76.7 ± 35.0
Ice cream and edible ice	225	224 (99.6)	18.7 ± 8.5	2.0	15.1	18.0	21.0	96.6	14.7 ± 8.9	0.0	10.9	13.3	17.0	96.6	76.5 ± 15.2
Milk	482	253 (52.5)	8.3 ± 11.2	0.0	3.8	5.0	7.9	61.6	3.3 ± 7.3	0.0	0.0	0.0	4.4	54.6	37.6 ± 41.7
Yoghurt and yoghurt drink	341	268 (78.6)	10.8 ± 6.2	0.0	7.4	11.1	13.2	57.0	6.3 ± 6.0	0.0	2.9	6.8	8.6	51.8	50.0 ± 28.8
**Edible oils and oil emulsions**	458	6 (1.3)	0.2 ± 1.3	0.0	0.0	0.0	0.0	26.1	0.6 ± 1.1	0.0	0.0	0.0	0.0	23.2	1.8 ± 11.9
Coconut oil	18	0 (0.0)	0.4 ± 1.5	0.0	0.0	0.0	0.0	6.3	0.0 ± 0.0	0.0	0.0	0.0	0.0	0.0	0.0 ± /[2]
Cooking oil spray	1	0 (0.0)	0.0 ± /	0.0	0.0	0.0	0.0	0.0	0.0 ± 0.0	0.0	0.0	0.0	0.0	0.0	/
Cooking oil	289	0 (0.0)	0.0 ± 0.0	0.0	0.0	0.0	0.0	0.1	0.0 ± 0.0	0.0	0.0	0.0	0.0	0.0	0.0 ± 0.0
Edible oil	150	6 (4.0)	0.6 ± 2.2	0.0	0.0	0.5	0.6	26.1	0.2 ± 1.9	0.0	0.0	0.0	0.0	23.2	1.8 ± 12.1
**Eggs**	71	3 (4.2)	0.6 ± 2.8	0.0	0.0	0.0	0.3	20.9	0.5 ± 2.8	0.0	0.0	0.0	0.0	20.9	11.5 ± 32.6
**Fish and fish products**	605	278 (46.0)	2.1 ± 3.9	0.0	0.0	0.6	3.2	60.2	1.9 ± 3.9	0.0	0.0	0.0	2.8	60.2	73.9 ± 44.0
**Fruits and vegetables**	2406	893 (37.1)	15.8 ± 21.8	0.0	1.0	4.7	20.4	89.4	8.2± 16.9	0.0	0.0	0.0	5.3	86.0	33.3 ± 43.7
Fruits (e.g. fruit bites, fruit bars)	445	228 (51.2)	41.2 ± 24.3	0.0	17.0	44.4	62.7	89.4	11.6 ± 17.8	0.0	0.0	1.0	16.6	86.0	29.7 ± 34.6
Herbs and spices	566	101 (17.8)	5.5 ± 9.8	0.0	0.0	1.6	7.2	71.6	2.6 ± 8.7	0.0	0.0	0.0	0.0	71.6	23.2 ± 41.9
Jam and Marmalades	217	206 (94.9)	48.3 ± 15.7	0.0	39.3	52.0	59.0	67.8	47.9 ± 16.8	0.0	39.3	52.0	59.0	67.8	94.9 ± 22.1
Nuts and seeds	427	97 (22.7)	6.8 ± 7.5	0.0	3.0	4.5	7.1	40.0	2.4 ± 6.8	0.0	0.0	0.0	0.0	37.2	11.7 ± 27.6
Vegetables	751	261 (34.8)	4.3 ± 7.4	0.0	0.2	2.2	4.8	79.5	2.1 ± 5.2	0.0	0.0	0.0	1.7	44.3	34.0 ± 45.5
**Meat and meat products**	660	487 (73.8)	2.6 ± 5.5	0.0	0.3	1.1	2.2	49.6	2.4 ± 5.5	0.0	0.0	0.9	2.1	49.6	80.2 ± 39.7
Meat Alternatives	102	36 (35.3)	1.5 ± 2.4	0.0	0.2	0.9	1.7	14.2	1.0 ± 2.4	0.0	0.0	0.0	1.1	14.2	40.1 ± 48,7
Processed meat	558	451 (80.8)	2.8 ± 5.9	0.0	0.3	1.2	2.3	49.6	2.6 ± 5.9	0.0	0.0	1.0	2.2	49.6	87.0 ± 33.6
**Non-alcoholic beverages**	2246	1498 (66.7)	12.5 ± 17.7	0.p	1.0	8.1	11.5	100.0	11.9 ± 17.3	0.0	0.0	8.0	11.4	100.0	89.9 ± 28.2
Beverage mixes	124	102 (82.3)	41.5 ± 27.6	0.0	18.1	42.6	59.8	100.0	38.1 ± 29.2	0.0	9.2	37.4	58.7	100.0	78.4 ± 36.8
Coffee and tea	627	227 (36.2)	11.8 ± 20.8	0.0	0.0	0.0	10.2	96.0	10.5 ± 20.0	0.0	0.0	0.0	7.8	96.0	64.7 ± 42.2
Cordials	93	90 (96.8)	42.2 ± 26.0	0.0	17.5	49.9	56.9	100.0	42.2 ± 26.0	0.0	17.5	49.9	56.9	100.0	100.0 ± 0.0
Electrolyte drinks	30	29 (96.7)	10.9 ± 22.2	0.0	4.2	5.9	6.6	92.5	10.9 ± 22.2	0.0	4.2	5.9	6.6	92.5	100.0 ± 0.0
Energy drinks	15	11 (73.3)	7.8 ± 6.1	0.0	0.0	9.1	13.8	15.5	7.8 ± 6.1	0.0	0.0	9.1	13.8	15.5	100.0 ± 0.0
Fruit and vegetable juices	523	399 (76.3)	9.8 ± 3.8	0.0	8.3	10.0	11.5	49.0	9.8 ± 3.8	0.0	8.3	10.0	11.5	49.0	100.0 ± 0.0
Soft drinks	667	610 (91.5)	8.5 ± 6.7	0.0	6.0	8.5	10.6	90.0	8.5 ± 6.7	0.0	6.0	8.5	10.6	90.0	100.0 ± 0.0
Waters (e.g. flavoured water)	166	30 (18.1)	2.0 ± 5.0	0.0	0.0	0.0	4.0	57.7	1.1 ± 4.9	0.0	0.0	0.0	0.0	57.7	36.7 ± 48.1

Major and minor food groups	Total *n*	*n* (%) containing FSI(s)	Total sugar content (g/100 g or g/100 mL)						Free sugar content (g/100 g or g/100 mL)						Free sugar as percent of total sugar ± SD (%)[1]
			Mean ± SD	Min	25th	50th	75th	Max	Mean ± SD	Min	25th	50th	75th	Max	
**Sauce, dressings, spreads and dips**	2207	1695 (76.8)	12.4 ± 14.2	0.0	2.6	5.7	18.0	80.7	10.9 ± 14.1	0.0	0.0	4.8	16.7	80.7	71.9 ± 39.9
Mayonnaise and salad dressings	326	267 (81.9)	12.4 ± 16.0	0.0	1.3	6.8	15.9	80.7	12.1 ± 16.1	0.0	0.0	6.7	15.9	80.7	89.3 ± 30.5
Sauce	1640	1245 (75.9)	11.9 ± 12.8	0.0	2.7	6.7	18.0	76.7	10.3 ± 12.9	0.0	0.0	4.5	16.7	76.7	70.8 ± 40.3
Spreads and dips	241	183 (75.9)	16.4 ± 19.0	0.0	3.1	8.3	25.0	67.1	13.3 ± 18.0	0.0	0.0	4.1	19.8	67.1	58.0 ± 40.5
**Snack foods**	981	780 (79.5)	10.3 ± 13.8	0.0	1.8	3.8	11.4	68.6	8.8 ± 12.9	0.0	0.3	3.4	9.7	66.0	82.5 ± 37.0
**Sugars, honey and related products**	487	470 (96.5)	77.7 ± 21.9	0.0	72.0	80.0	96.0	100.0	77.4± 22.5	0.0	72.0	80.0	96.0	100.0	98.7 ± 11.2
Dessert additions	9	9 (100.0)	63.7 ± 21.7	40.0	42.9	61.7	84.5	98.5	63.7 ± 21.7	40.0	42.9	61.7	84.5	98.5	100.0 ± 0.0
Dessert toppings	37	34 (91.9)	59.8 ± 26.0	4.5	46.3	57.1	86.3	100.0	59.3 ± 27.0	0.0	46.3	57.1	86.3	100.0	91.9 ± 27.7
Honey	195	195 (100.0)	76.4 ± 6.6	47.0	73.4	78.1	81.0	88.9	76.4 ± 6.7	47.0	73.4	78.1	81.0	88.9	99.9 ± 0.7
Sugar	156	156 (100.0)	94.8 ± 12.4	0.0	94.1	99.0	99.9	100.0	94.8 ± 12.4	0.0	94.1	99.0	99.9	100.0	100.0 ± 0.0
Sweeteners	24	11 (45.8)	34.0 ± 43.6	0.0	0.0	4.9	92.0	100.0	31.6 ± 44.7	0.0	0.0	0.0	92.0	100.0	84.6 ± 37.6
Syrup	66	65 (98.5)	68.8 ± 16.4	23.3	59.5	73.6	80.0	100.0	68.3 ± 17.9	0.0	59.5	73.6	80.0	100.0	98.5 ± 12.3
**Total**	18784	12118 (64.5)	15.8 ± 22.0	0.0	1.2	5.7	21.4	100.0	13.3 ± 21.2	0.0	0.0	3.2	17.2	100.0	65.8 ± 43.4

[1]Free sugar percentage was calculated (free sugar/total sugar content) for each product. The average free sugar percentage of each food category was shown here.
[2]Only one product has a total sugar content larger than 0 g in this category, therefore a SD is not applicable. Abbreviation: FSI = free sugar ingredient.
